# MicroRNA as an Early Biomarker of Neonatal Sepsis

**DOI:** 10.3389/fped.2022.854324

**Published:** 2022-05-09

**Authors:** Martin Jouza, Julia Bohosova, Andrea Stanikova, Jakub Pecl, Ondrej Slaby, Petr Jabandziev

**Affiliations:** ^1^Department of Pediatrics, University Hospital Brno, Brno, Czechia; ^2^Faculty of Medicine, Masaryk University, Brno, Czechia; ^3^Central European Institute of Technology, Masaryk University, Brno, Czechia; ^4^Department of Neonatology, University Hospital Brno, Brno, Czechia; ^5^Department of Biology, Faculty of Medicine, Masaryk University, Brno, Czechia

**Keywords:** miRNA, inflammation, CRP, IL-6, sepsis

## Abstract

Sepsis is a major cause of lethality in neonatal intensive care units. Despite significant advances in neonatal care and growing scientific knowledge about the disease, 4 of every 10 infants born in developed countries and suffering from sepsis die or experience considerable disability, including substantial and permanent neurodevelopmental impairment. Pharmacological treatment strategies for neonatal sepsis remain limited and mainly based upon early initiation of antibiotics and supportive treatment. In this context, numerous clinical and serum-based markers have been evaluated for diagnosing sepsis and evaluating its severity and etiology. MicroRNAs (miRNAs) do not encode for proteins but regulate gene expression by inhibiting the translation or transcription of their target mRNAs. Recently, it was demonstrated in adult patients that miRNAs are released into the circulation and that the spectrum of circulating miRNAs is altered during various pathologic conditions, such as inflammation, infection, and sepsis. Here, we summarize current findings on the role of circulating miRNAs in the diagnosis and staging of neonatal sepsis. The conclusions point to substantial diagnostic potential, and several miRNAs have been validated independently by different teams, namely miR-16a, miR-16, miR-96-5p, miR-141, miR-181a, and miR-1184.

## Introduction

The term neonatal sepsis is used to label systemic inflammation of bacterial, viral, or fungal origin associated with hemodynamic changes and other clinical manifestations leading to significant morbidity and mortality in newborns. Traditionally, the definition of sepsis has included isolation of the pathogen from usually sterile body fluid (blood or cerebrospinal fluid) along with the definition of systemic inflammatory response syndrome (SIRS) (1). Sepsis is the third most common cause of death among neonates, accounting for 225,000 deaths globally every year (2).

In 1990, both the United Nations and World Health Organization (WHO) prioritized a two-thirds reduction by 2015 in the unacceptably high child mortality rate. Nonetheless, 2.3 million children under 5 years of age died in 2013 from infectious diseases (708,600 deaths were caused by lower respiratory infection, 474,900 deaths by diarrhea, and 570,000 deaths by malaria) (3). Increasing from 37% in 1990, 44% of deaths in children under 5 years of age occurred during the neonatal period, again predominantly due to infectious causes, including sepsis. In 2017, WHO classified sepsis as a critical healthcare priority for the coming decade (4).

Despite significant advances in neonatal care and growing scientific knowledge about the disease, 4 of every 10 infants born in developed countries and suffering from sepsis die or experience considerable disability, including substantial and permanent neurodevelopmental impairment (1, 5). Sepsis in The Global Burden of Disease Study from 2013 is classified as the second-leading cause of death in neonates. This work reports 342,200 deaths from neonatal sepsis globally (6).

Fleichmann-Struzek et al. published an extensive systemic review analyzing the global burden of pediatric and neonatal sepsis. The review was based upon 1,270 studies published from 1979 to 2016. The population-level estimate for neonatal sepsis was 2,202 (95% confidence interval 1,099–4,360) per 100,000 live births, with mortality between 11 and 19%. Extrapolating these figures on a global scale, they estimated an incidence of 3.0 million cases of sepsis in neonates annually (7).

Prematurely born neonates experience the highest incidence and mortality of sepsis among all age groups. Compared to term infants, sepsis in preterm infants is as much as 1,000-fold more common and is associated with higher mortality rates and life-long neurodevelopmental handicaps (8). Notably, it is estimated that 11% (approximately 15 million babies) of the 135 million births globally occur before 37 weeks of completed gestation (preterm). The incidence of preterm births has been increasing steadily, especially in developed countries. With 1 million children dying due to preterm birth before 5 years of age, preterm birth is the leading cause of death among children, accounting for 18% of all deaths among children aged under 5 years and as much as 35% of all deaths among newborns (aged <28 days) (9).

Neonatal sepsis has been classified according to the age of onset and timing of sepsis episode as either early-onset sepsis (EOS) or late-onset sepsis (LOS). Clinical manifestation of EOS is usually within the first 72 h of life (10). Some clinicians regard group B streptococci infection in the first 7 days as early-onset sepsis. EOS is an infection caused by a pathogen transmitted vertically from mother to infant before or during labor. LOS sepsis, on the contrary, is an infection occurring after 72 h or seven days, respectively. LOS is connected to a community or hospital pathogens (11).

Standard laboratory tests and clinical measurements (i.e., I/T index, acute phase proteins, and heart rate) in sepsis are limited in their diagnostic accuracy, and especially they have low positive predictive value (12, 13). These limitations, combined with vague clinical signs overlapping with the signs of general immaturity of neonates, push neonatologists to rule out sepsis too early (1). In case of delaying needed antibiotic therapy, the complete symptoms of sepsis could develop, and the clinical outcome could be worsened (14). Of course, too-liberal antibiotic policies also are not desirable and lead to increased microbial resistance (15).

Improving the diagnosis of neonatal sepsis must be one of the essential aims of modern medicine. Despite our best effort, neonatal sepsis remains a burden and source of considerable mortality, organ dysfunction, and high hospitalization costs. Therefore, the discovery of rapid and accurate biomarkers for diagnosis and prognosis estimation in neonatal sepsis should be given high priority (16).

## Biomarkers of Sepsis

The National Institute of Health defines a biomarker as “a characteristic that is objectively measured and evaluated as an indicator of normal biological processes, pathogenic processes, or pharmacologic response to a therapeutic intervention” (17). Analysis of biomarkers should theoretically lead to confirmation or rejection of the sepsis diagnosis and help estimate the prognosis of individual patients. Blood culture has been the gold standard in differentiating infection and non-infection conditions despite its being time-consuming and yielding an enormous amount of false-negative results (18). In the past, blood components have been routinely analyzed as, for instance, the low platelet count is one of the typical laboratory manifestations of sepsis and the severity of thrombocytopenia correlates with the severity of infectious disease (19).

Various biomarkers, such as levels of C-reactive protein (CRP), procalcitonin (PCT), or interleukin-6 (IL-6), have been implemented in the past to diagnose suspect sepsis. Most of these are associated with inflammation generally, and thus their specificity for infection is low and could be affected by many other reasons, such as burns and malignant diseases in the case of CRP (20). Recent meta-analyses have shown low sensitivity and specificity of CRP (0.75 and 0.67) as proof of infection (21). On the other hand, CRP level correlates with the severity of the condition (22), and its decrease during sepsis therapy correlates with the effectiveness of the treatment (23). Elevated PCT is more likely associated specifically with sepsis. Hence, PCT is compared to CRP as a part of the official guideline for diagnosing sepsis and infection in adult medicine (24). PCT is a calcitonin prohormone that in healthy individuals is secreted only by the thyroid gland's neuroendocrine cells. During infection, however, PCT is secreted from cells in almost every organ (25). A meta-analysis has confirmed that PCT is relevant for evaluating antibiotic therapy's effectiveness in septic patients (26). The pro-inflammatory cytokine IL-6 also has been used in diagnosing sepsis. IL-6 is secreted by naïve immune cells (monocytes, phagocytes, T cells, B cells), thereby activating acute-phase proteins. IL-6 levels correlate well with the severity of sepsis and could be used as a prognostic biomarker (27, 28). Additionally, several novel molecules are currently being studied as potential biomarkers in sepsis. MCP-1 (monocyte chemoattractant protein-1), PD-1/PD-L1 (programmed death receptor-1, a programmed death ligand-1), sTREM-1 (soluble triggering receptor expressed on myeloid cells-1), CD64 (neutrophil surface receptor), sCD14-ST (presepsin), and finally microRNAs are other promising but not widely used biomarkers of sepsis (29).

## Role of microRNAs in the Development of Sepsis

**Figure 1:**
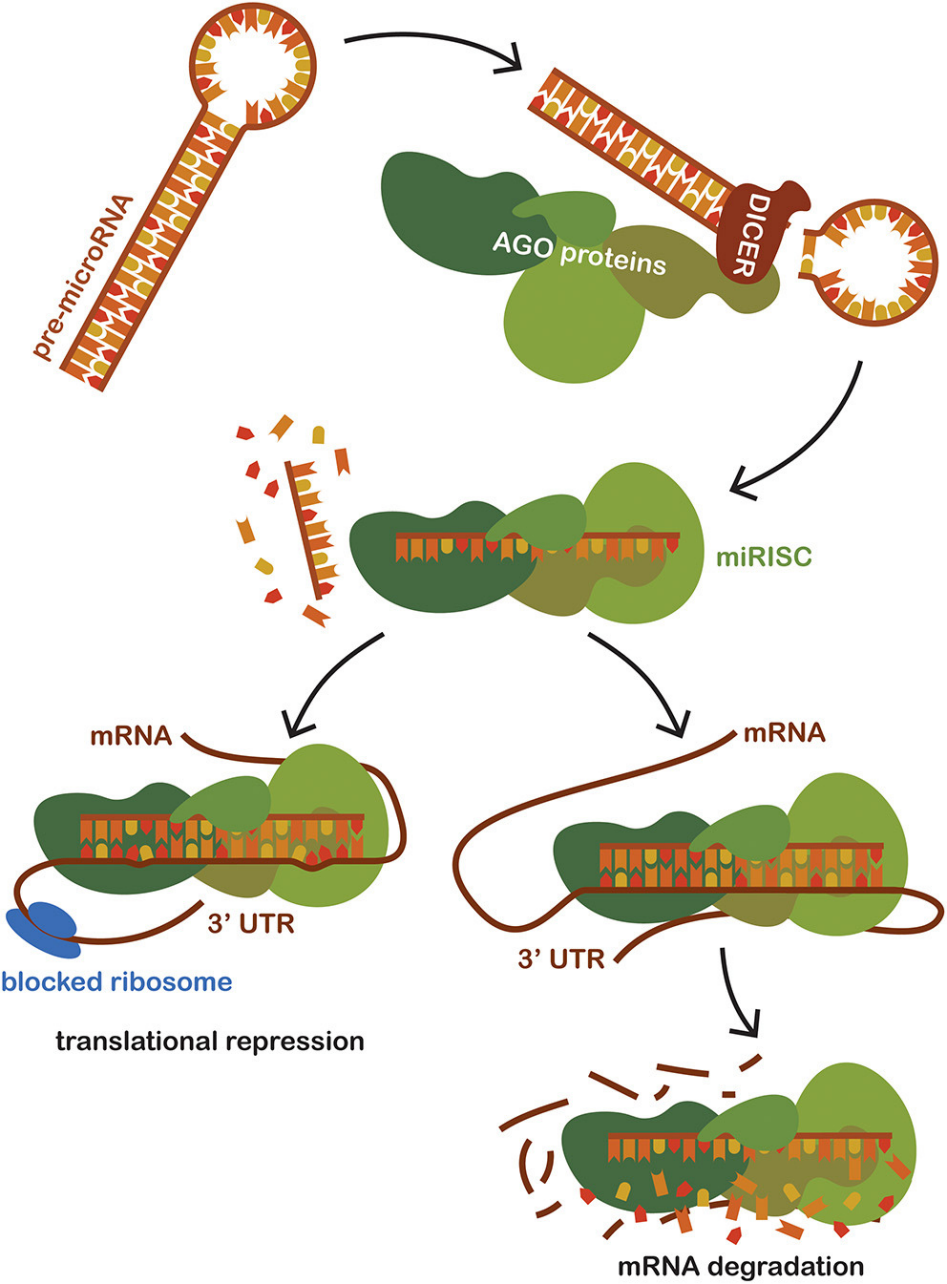
Schematic representation of miRNA mechanism of action. Pre-miRNAs are exported to the cytoplasm from the nucleus and then processed by the Dicer (RNase III endonuclease). Dicer removes the terminal loop, and a mature miRNA is formed. Mature miRNA joins the Argonaut protein family (AGO), creating a miRNA-induced silencing complex (miRISC) which subsequently binds to the target mRNA. Targeting is based on the sequence complementarity between the 3'untranslated region of mRNA and the seed region of miRNA incorporated in the miRISC. The binding of miRISC leads to the destabilization of the target miRNA and repression of translation, thus impacting the amount of the final translated protein product of a given gene.

MicroRNAs (miRNAs) is the name for a group of hairpin-derived non-coding transcripts approximately 20–24 nucleotides long and acting as post-transcriptional regulators of gene expression. Currently, we distinguish more than 2,300 distinct miRNAs in the human genome (30), many of them strongly conserved among different species. They are produced endogenously and expressed from introns of protein-coding genes or from specific miRNA genes creating a primary hairpin transcript, pri-miRNA, which is further cleaved and processed first in the nucleus and then transported and matured in the cytoplasm. Pre-miRNAs are exported to the cytoplasm and then processed by the Dicer (RNase III endonuclease). The removal of the terminal loop results and a mature miRNA being formed, then a short double-stranded structure occurs (31). One of the strands is incorporated into a complex with Argonaute family proteins (AGO) to create a so-called miRNA-induced silencing complex (miRISC) that primarily facilitates mRNA destabilization or degradation. The miRNA strand serves as a target recognition guide (21, 32, 33). The mechanism of action is based upon binding to the 3′UTR region of the target mRNA, regulating more than 60% of all protein-coding genes in humans (34). The mechanism of action of miRNA shows in Figure 1. Such a scope is possible due to their pleiotropic specificity, as the binding is facilitated only by the small extended seed region of the miRNA sequence consisting of eight nucleotides. The miRNA mechanism of gene expression regulation is deeply conserved. Although their effects are subtle, miRNAs are involved in the fine-tuning translation of a wide number of mRNAs available for translation (35). Although initially discovered and studied in cancer (32, 36, 37), miRNA levels dysregulation has since been observed in many other human conditions and diseases, suggesting that miRNA targeting presents a novel therapeutic approach, one that is effective, for example, in epilepsy (38–41). Moreover, the exceptional stability of these short molecules and their spatiotemporal specificity predestines them to be the much-sought biomarker for sepsis as well as in other human conditions (42).

In addition to their other vital roles in humans, miRNAs play important roles in specific and non-specific immune system responses to various pathogenic states, such as atherosclerosis, rheumatoid arthritis, diabetes mellitus, and bacterial infections (43). Recently, it has been confirmed that miRNAs regulate the immune system and its development, including the differentiation and function of immune cells (44, 45). Various miRNAs directly target the signaling pathway of tumor necrotizing factor (TNF), one of the major regulators of pro-inflammatory processes during sepsis (46–48). Increased expression of miR-155 has been detected in macrophages, and liver cells after bacterial lipopolysaccharide (LPS) stimulation (49), as well as have been miR-150 and let-7a in an LPS-induced monocytic cell line (50). The toll-like receptor (TLR)/NF-κB signaling pathway is another area of miRNA-mediated regulation. TLR is a part of innate immunity that mediates the systemic inflammatory response to pathogens during sepsis (51). Expressed on macrophages, dendritic cells, and various antigen-presenting cells, TLR interacts with PAMPS (pathogen-associated molecular patterns), produced by bacterial pathogens, or DAMPS (danger-associated molecular patterns), released from necrotic or dying cells. We currently distinguish ten human TLRs. The bacterial LSP explicitly activates TLR4. Therefore, TLR4 has a fundamental role in initiating the septic state (52).

Based upon the robust evidence as to the involvement of miRNAs in immune regulation, inflammatory response, and development of sepsis, efforts to harness their biomarker potential have increased in recent years. For example, Vasilescu and his team determined a lower level of miR-150 in the serum of 16 septic adult patients, and those low levels correlated with high levels of pro-inflammatory markers (53). Moreover, the serum level of miRNA could be used for the identification and prognosis of sepsis (54–56). Studies on adult patients prove an association between specific expression levels of several miRNAs and the death of septic patients (57). In their extensive 2016 review, Benz et al. report miRNAs with diagnostic potential concerning sepsis in adult patients (21). Yao et al. imply in their work that miR-25 could be utilized not only as a diagnostic but also as a prognostic biomarker (58). MiR-122 is part of a six-microRNA panel (miR-223, miR-15a, miR-16, miR-122, miR-193, and miR-483-5p) that could predict short-term and long-term sepsis survival with high accuracy (59). Furthermore, an elevated level of miR-122 is associated with poor neurological outcomes in patients after cardiac arrest, with chronic liver disease, and with hepatocellular carcinoma (21). Rau et al. studied miR-133a along with a panel of another nine upregulated microRNAs (miR-133a-1-3p, miR-133-2-3p, miR-133a-1-5p, and miR-133b-3p). High levels of miR-133a indicate poor survival and could be used as an independent indicator of clinical outcome (60).

## MicroRNAs as Promising Biomarkers of Neonatal Sepsis

Recent studies suggest a massive potential for miRNAs as prognostic and diagnostic biomarkers in adult sepsis. These results most certainly cannot, however, be directly extrapolated to neonatal patients with sepsis due to the widely different conditions, age, developmental stage, and overall state of the organism struck by neonatal sepsis. Using Boolean operators, we searched the Pubmed database for keywords “neonatal sepsis” AND “microRNA.” Out of 21 search results, we excluded unrelated papers and further worked only with those concerning patients with neonatal sepsis and miRNA expression profiling or functional *in vitro* tests regarding their role in neonatal sepsis. Sixteen studies remained for further consideration and are summarized in Table 1.

**Table 1:** Summary of original research articles on miRNA dysregulation in patients with neonatal sepsis.

**References**	**Studied material (N of patients, controls, material)**	**Explorative part (method)**	**Significant dysregulation (qPCR validation)**	**Change in expression**	***P*** **-value**
Huang et al. (46)	Neonatal umbilical cord blood and adult peripheral blood—separated MNC, LPS induced	Illumina BeadArray	miR-125b miR-130a miR-155	Down (all after LPS treatment)	<0.05 (all)
Chen et al. (61)	24 septic neonates/24 healthy newborns; peripheral blood—leukocytes	Microarray (miRCURRY LNA microRNA Array)	miR-29a miR-96 miR-141 miR-181a miR-1184 miR-101 miR-185	Down Down Down Down Down Up UP	<0.0001 <0.001 0.014 0.045 0.015 0.02 0.001
Wang et al. (62)	46 septic neonates 41 RI neonates, Peripheral blood	–	miR-15a miR-16	Up Up	<0.0001 <0.0001
Huang et al. (63)	Neonatal umbilical cord blood and adult peripheral blood—separated PMN, LPS-induced	Illumina BeadArray	miR-26b miR-142-3p let-7g	Down Down Down	<0.05 (all)
Huang et al. (64)^*^	17 septic neonates, 19 healthy controls	Microarray—online dataset	–	–	–
Dhas et al. (65)	25 septic neonates, 25 healthy controls, plasma	–	miR-132 miR-223	Down Down	<0.05 <0.01
Li et al. (66)	Cell culture, mouse model	–	miR-300	Down	–
Wang et al. (67)	32 septic neonates, 30 RI neonates, serum	–	miR-15a miR-16	Up Up	0.008 0.002
Cheng et al. (68)	28 septic neonates, 32 healthy controls, Serum and MNC	–	miR-26a	Down	<0.01
Ng et al. (69)	232 suspected sepsis/NEC patients, plasma	Microarray	miR-1290 miR-1246 miR-375	Down Down Down	<0.001 <0.001 0.003
Liu et al. (70)	102 septic neonates, 50 RI controls, serum and separated MNC, LPS-induced	–	miR-181a	Down	<0.01
Chen et al. (71)	30 septic neonates, 24 RI neonates	–	miR-96-5p	Down	<0.05
El-Hefnawy et al. (72)	25 septic neonates, 25 healthy controls, serum	–	miR-16a miR-451	Up Up	<0.001 0.034
Lin and Wang (73)	98 septic neonates, 50 RI controls, serum and separated MNC, LPS-induced	–	miR-141	Down	<0.001
Fouda et al. (74)	25 septic neonates, 25 healthy controls, serum	–	miR-15b miR-378a	Up Down	<0.001 <0.001
Wang and Han (75)	72 septic neonates, 56 RI controls, serum and separated MNC, LPS-induced	–	miR-1184	Down	<0.001

*MNC, mononuclear cells; NEC, necrotizing enterocolitis; RI, respiratory infection*.

* *miR-150 identified as sepsis-associated by analysis of the online dataset, no validation of the results provided*.

The first paper focused on miRNA and concerning the development of sepsis was published in 2012 (46). It suggested that miR-125b is involved in the regulation of TNF-α production in neonatal monocytes activated with LPS. Illumina BeadArray was used to explore miRNA expression. It detected 470 miRNAs significantly dysregulated. Candidate miRNAs were subsequently validated by quantitative polymerase chain reaction (qPCR). As a greater expression of TNF-α was observed in neonatal monocytes, a further investigation focused on miRNAs with TNF-α mRNA binding sites. Not only was the expression level of miR-125b lower and that of TNF-α higher in neonatal monocytes after LPS treatment, but the artificial increase of miR-125b also led to a decrease in TNF-α. That supports the notion that miR-125b is involved in the regulation of TNF-α expression. Aside from miR-125b, miR-130a also reacted to LPS stimulation. Meanwhile, miR-155 was higher in adult monocytes. Continuing to work with LPS-induced inflammation in white blood cells, the same team showed that IL-6 mRNA and protein expression was higher in neonatal polymorphonuclear monocytes (PMN) than in adult PMN. After further inspection, levels of miR-26b, miR-142-3p, and let-7g were altered in neonatal PMN after LPS induction compared with adult PMN. Moreover, the artificial increase of miR-142-3p and let-7g led to the repression of IL-6 expression. This result suggested that an impaired inflammatory reaction of neonates with generally increased levels of IL-6, which might contribute to disease morbidity, also involves dysregulation of critical miRNA regulators of IL-6 expression.

In the work of Cheng et al. the strategy was similar. After initial explorative profiling using microarrays, the dysregulated miRNAs related to the immune response and inflammation were further studied. This time, however, theirs was the first work to compare septic and healthy neonates and thus to be designed as a conventional biomarker study. The authors identified significant dysregulation of expression in several miRNAs associated with more than 60 immune-related genes. Moreover, these miRNAs had not previously been associated with sepsis. As the authors note, the set of miRNAs identified as dysregulated in this study differs significantly from miRNAs typically dysregulated in adults with sepsis. That underscores the specific immune status of neonates. Although promising, it should be noted that the same cohort was used for both the exploration and validation, thus limiting the statistical value of the results (61).

Wang et al. found that miR-15a and miR-16 are potential diagnostic biomarkers of sepsis. In their study, they chose a panel of inflammation-associated miRNAs and measured them using qPCR. Both miRNAs were found to be increased in septic patients, thus suggesting their diagnostic potential. Moreover, upregulation of both miRNAs was associated with decreased expression in RAW264.7 cells of TLR4 and IRAK-1, both of which are crucial players in LPS-induced inflammatory response (62).

Another team has provided further evidence regarding the role of miR-15a and miR-16 in sepsis. Moreover, these authors discovered an interplay with long non-coding RNA SNHG16 creating a regulatory network. SNGH16, in this case, serves as a so-called competing endogenous RNA (ceRNA), a sponge binding complementary miRNAs and thus reducing their availability for further regulation of their target mRNA. Moreover, contrary to levels of miR-15a and miR-16, levels of TLR4 were downregulated along with the levels of SNHG16 in serum. As SNHG16 binds miR-15a and miR-16, which in turn cannot bind to TLR4 mRNA, lower levels of SNHG16 leave more miRNA molecules in the pool, which further lowers the levels of TLR4 protein (67).

Some studies suggest that miR-300 targets nicotinamide phosphoribosyltransferase (NAMPT), one of the primary regulators of an inflammatory response, through activation of the AMP-activated protein kinase/mammalian target of rapamycin (AMPK/mTOR) pathway (66). Using complex *in vitro* tests, Li et al. revealed that miR-300 targets NAMPT, thus activating the AMPK/mTOR signaling pathway and autophagy. Moreover, miR-300 overexpression of NAMPT silencing leads to CD1 cell cycle progress and reduction of apoptosis (66).

The regulatory role of miR-26a has been investigated by Cheng et al. Expression levels of miR-26a in the serum of neonatal patients with sepsis were found to be significantly lower than those in healthy non-septic controls. The tests in this study on LPS-induced monocytes showed direct regulation of IL-6 expression by miR-26a binding. Thus, lower levels of this miRNA in septic patients lead to higher levels of IL-6, which in turn potentiates inflammatory response in many immune cells, such as monocytes, phagocytes, T and B cells, fibroblasts, and others. Activating these cells by heightened IL-6 signaling is known to lead to the pathogenesis of inflammatory diseases such as sepsis (68).

The most extensive study on this topic to date has been provided by Ng et al. who profiled miRNA expression in 232 suspected sepsis or necrotizing enterocolitis (NEC) cases. Although the primary goal was to identify diagnostic biomarkers for NEC, as its distinction from sepsis is problematic, the authors compared the NEC group to other non-septic/non-NEC infants diagnosed with other diseases and healthy infants without health problems. miR-1290, miR-1246, and miR-375 were identified as significantly downregulated in septic patients compared to NEC cases. These miRNAs were generally downregulated also in healthy and non-NEC/non-septic controls, however, suggesting that these miRNAs are specific for NEC rather than being a suitable biomarker also for neonatal sepsis (69).

Regarding the expression levels of miR-181a, Liu et al. (70) looked deeper into this miRNA's involvement in the development of sepsis. The level of miR-181a was significantly lower in the serum of septic patients compared with non-septic controls, similarly to Chen et al. (61). The expression was also lowered in LPS-induced monocytes compared to untreated cells. Moreover, miR-181a directly regulated TLR4 in monocytes, as well as inflammatory cytokines TNF-α and IL-8.

Additional partial validation of the results from Chen et al. has been provided recently. Downregulation of miR-96-5p has been validated, and functional *in vitro* tests showed that miR-96-5p directly and negatively regulates inflammatory response in LPS-induced cells by regulating TNF- α, IL-6, NAMPT, and the NF-κB pathway (71). Lastly, Wang et al. have shown significant downregulation of miR-1184 in septic neonates and correlation with levels of serum pro-inflammatory cytokines Il-1β, IL-6, and TNF-α. Artificial overexpression of this miRNA improved inflammatory response in LPS-induced monocytes (75).

Currently available studies rarely provide receiver operating characteristic (ROC) analysis of their results. Therefore, it is difficult to draw any conclusions regarding the biomarker potential of the studied miRNAs. There are some exceptions, however. Lin et al. showed that miR-141, with an area under the ROC curve (AUC) of 0.87, has tremendous diagnostic potential. That improves to AUC 0.93 when combined with serum levels of procalcitonin. This miRNA is also involved in the regulation of TLR4 in LPS-induced monocytes and pro-inflammatory cytokines TNF-α and IL-8 (73). The work of Fouda et al. shows excellent diagnostic properties of miR-15b and miR-378a (AUC 0.965, 0.952). While miR-15b was upregulated, miR-378a was downregulated in septic neonates compared to healthy controls (74). A recent study from El-Hefnawy et al. shows the diagnostic potential of miR-16a, which, with AUC 0.968, is significantly upregulated in septic patients compared to healthy controls (72).

In general, current results suffer from insufficient use of high-throughput profiling methods, especially RNAseq, which could help to discover novel miRNAs associated specifically with neonatal sepsis. Most of the studies were based upon a hypothesis-driven approach and were focused only on a select few miRNAs with the premise of their involvement in inflammation. At the same time, the comparison is usually made with healthy controls. This, then, raises the question of whether these miRNAs are specific for sepsis or, instead, for inflammation more broadly. Therefore, enrolling other infectious patients as a control cohort is advisable, as was done in several reviewed studies (67, 69–71, 73, 75). Moreover, although some independent validation of the results by other authors has been achieved in cases of miR-16a, miR-16, miR-96-5p, miR-141, miR-181a, and miR-1184, individual studies rarely include global expression profiling and qPCR validation on independent cohorts. Larger cohorts and independent confirmation of the results are crucial, however, for any meaningful and robust biomarker determination.

In addition, current research lacks prospective studies with data and sample collection even before the sepsis symptoms occur. This almost surely would help to identify early sepsis biomarkers that would allow for intervention and vigilance in high-risk neonates. The miRNA diagnostic potential is nevertheless evident, and it should be investigated further for future use in earlier and more precise diagnostic and effective medicinal interventions. A separate task is the determination of predictive biomarkers, which have not been investigated to date, as all those articles reviewed focused solely on the distinction between septic and non-septic neonates. The discovery of molecules predicting the clinical outcome and severity of the disease would bring even more precious information for clinicians.

## Conclusion

Early diagnosis of neonatal sepsis is still a very important goal of modern medicine, and this is routine clinical work nearly every day in neonatal intensive care units. The gold standard in diagnostics of neonatal sepsis—isolating the causative agent from a normally sterile body site (e.g., blood, CSF, urine, as well as pleural, joint, and peritoneal fluids)—remains time-consuming and unspecific. An additional tool that helps in establishing a diagnosis is the widely used assessment of serum biomarkers (CRP, PCT, IL-6). Those biomarkers currently in use, however, are associated with general inflammation and are easily misleading due to the general immaturity of the neonatal organism and its higher irritability. While sepsis poses a significant problem in clinical research due to its considerable incidence in neonates, the search for early and precise diagnostic and prognostic biomarkers is beginning newly to bring to the fore molecules potentially feasible in clinical practice, namely miRNAs. Current evidence points to their strong diagnostic potential, specifically in the cases of several miRNAs independently validated by different teams, those being miR-16a, miR-16, miR-96-5p, miR-141, miR-181a, and miR-1184. Nevertheless, high-throughput profiling methods and validation on an independent cohort of patients are necessary to produce more robust evidence (e.g., standardization for miRNA quantification assays, standardization of the different testing materials, more precise defining of preanalytical and analytical conditions). Moreover, prospective samples and data collection before the disease's onset and serial samples during sepsis would bring better insight into the possibility for early diagnosis of sepsis.

## Author Contributions

MJ and PJ: conceptualization. MJ: methodology. MJ, AS, and JB: writing—original draft. JB: writing—review and editing. OS and PJ: supervision and funding acquisition. All authors contributed to the article and approved the submitted version.

## Funding

This work was supported by the Ministry of Health, Czech Republic - conceptual development of research organization (FNBr 65269705).

## Conflict of Interest

The authors declare that the research was conducted in the absence of any commercial or financial relationships that could be construed as a potential conflict of interest.

## Publisher's Note

All claims expressed in this article are solely those of the authors and do not necessarily represent those of their affiliated organizations, or those of the publisher, the editors and the reviewers. Any product that may be evaluated in this article, or claim that may be made by its manufacturer, is not guaranteed or endorsed by the publisher.
